# Sporadic Typhoid

**Published:** 1903-02-07

**Authors:** 


### Sporadic Typhoid.

The evidence which has been steadily accumulating during the past few years as to the conveyance of enteric fever by shell fish seems to give a clue to the causation of many of those sporadic cases of the disease which have always been somewhat of a puzzle to the epidemiologist. It may be well here to recall the fact that until, by the careful observation of a series of outbreaks of water-typhoid, the specific nature of the disease had been proved, there was much to be said in favour of the view that amid filthy surroundings the infection might arise *de novo*. One of the many names by which enteric fever has been described—that of pythogenic fever—was specially intended to express this view, viz., that it was a filth-bred fever. Under these circumstances there was then nothing either puzzling or surprising in the occurrence of isolated or sporadic cases.

When, however, we had become convinced that enteric fever was due to the swallowing of an infective material derived from a preceding case of the disease the sporadic case became somewhat of a stumbling block. Easy as it had seemed while the doctrine of spontaneous generation held an uncertain sway over men's minds to believe in pythogenic fever, it was no longer easy to accept this origin for a fever the lesions of which were distinctly specific, and the infection of which, if accidentally distributed, was capable, as had been proved over and over again, of setting up in endless succession precisely similar fevers having precisely similar characteristics. Hence, to explain the sporadic case, we had to invent the theory that the infection could remain latent in the soil, growing, or at least maintaining its vitality, during long periods, and then when at last washed by rain into some water-supply and swallowed by some luckless individual taking on again its former virulence as a pathogenic organism. In its own proper place this theory is probably true. The results of many very careful investigations go to prove that without doubt the typhoid organism, if not gobbled up by other competitors in the struggle for existence, can maintain its vitality for considerable periods, and can ultimately resume its virulence, and there seems much reason to believe that this is the explanation of some of those curious instances in which at comparatively long intervals, without any known fresh introduction of infection, cases of typhoid fever crop up time after time on the same farm or in the same hamlet. In towns which are provided with a good water supply, however, the sporadic case has often been difficult to explain. Flies, dust-bins, defective methods of conservancy, etc., often give the clue to an undue prevalence of typhoid in certain courts or districts, but these are not truly sporadic cases. They do not arise apparently *de novo*; they do not subside leaving apparently no abiding mischief, as so often happens in true sporadic typhoid. To explain what is constantly happening in this regard we must imagine an infection imported absolutely from the outside and one capable of setting up the disease in persons whose surroundings are of a satisfactory nature, as is shown by the disease spreading no further; and when it is asked, what sort of an infection can this be? the answer is shell-fish—cockles, or oysters which have been laid in sewage-polluted water.

A moment's consideration is sufficient to show what a toss up it is whether an oyster so laid does or does not become a vehicle of infection. Sewage does not consist of a homogeneous liquid throughout the whole of which its various constituents are evenly and impartially distributed. It consists of a fluid in which float a large number of bits of solid matter, no two of which are alike in regard to the infection with which they may or may not be charged. Into the open shell of one oyster there may float a fragment charged with virulent infection, while into the shells of ten thousand others there may enter nothing of any moment. Thus the distribution of infection is an absolute lottery. Then turn to the drawing of this lottery. It may so happen, in fact it has happened on more than one occasion, that soon after the collection of a quantity of infected oysters some great feast has been held to which these dainties have been sent, and at which they have been freely eaten, with the result that an "outbreak" of typhoid has taken place of sufficient intensity to draw attention at once to its cause. But in ninety-nine cases out of a hundred no such thing occurs; the lottery is drawn in dozens of different restaurants, at scores of different oyster stalls, and in many different towns. Out of the thousands of oysters sold, only one here and there may have received its infection in such quantity and at such a date as to be capable of acting injuriously at the moment it is swallowed, and thus it happens that in the great majority of cases "shell-fish typhoid" is spread haphazard over large areas, arising apparently without cause, sometimes acting as the starting-point of local outbreaks, sometimes ending with the single case. So far as present knowledge enables one to speak positively at all, there seems no doubt that we must regard the consumption of infected shell-fish as the explanation of many apparently inexplicable cases of sporadic typhoid.

